# Inpatient gradual diagnostics and its relevance for determining treatment strategies in lumbar back pain

**DOI:** 10.1186/s12891-016-1153-1

**Published:** 2016-07-12

**Authors:** Ulf Krister Hofmann, Marco Gesicki, Falk Mittag

**Affiliations:** Department of Orthopaedic Surgery, University Hospital of Tübingen, Hoppe-Seyler-Strasse 3, D-72076 Tübingen, Germany; Praxis Dres. Falck and Gesicki, Horemer 4, D-72076 Tübingen, Germany

**Keywords:** Clinical stepwise diagnosis, Inpatient gradual diagnostics, Lumbar back pain, Lumbar infiltration, Lumbar spine surgery

## Abstract

**Background:**

Identifying patients who will benefit from spine surgery is still a challenge. This is especially the case when patients’ complaints and medical history, together with clinical observations, do not correspond to structural pathological changes. With inpatient gradual diagnostics (IGD)—the administration of analgesic and anti-inflammatory agents to a special area of interest—the effect of surgery can be temporarily simulated. From the patient’s statement about the alleviation of pain, the surgeon can draw conclusions concerning its causes. The aim of this study was to evaluate the extent to which IGD influences the decision about the nature and scope of surgical treatment strategies, and the way in which it does so, in patients with chronic lumbar back pain.

**Methods:**

Clinical history and radiologic images were analysed retrospectively in 116 patients by two spine surgeons. Two therapeutic recommendations were developed for each patient: one was based on knowledge before IGD and one on knowledge after IGD.

**Results:**

IGD changed the treatment strategy in 39 % of the analysed cases. Although the rate of recommended surgery was reduced by about 10 %, the indicated surgical scope increased in 25 % of cases.

**Conclusions:**

IGD is an established concept used to determine therapeutic strategies in patients with chronic lumbar back pain. In our analysed cases, IGD led to highly relevant changes in recommendations for further surgical treatment.

**Electronic supplementary material:**

The online version of this article (doi:10.1186/s12891-016-1153-1) contains supplementary material, which is available to authorized users.

## Background

Lumbar back pain, with a lifetime prevalence of about 80 %, leads to enormous direct and indirect costs to the health care system [1–3]. It can affect people of all ages and, once ocurred, in many cases is a recurrent condition [4]. While benign and mixed back pain are believed to reach their maximum in the sixth decade of life, it is assumed that severe disabling episodes due to chronic low back pain rise in prevalence with increasing age [5, 6]. Depending on the cause of the patient’s complaints, as well as the patient’s condition and expectations, therapeutic approaches range from conservative treatment, including physiotherapy with analgesics, orthotics, and local infiltration therapy, to multimodal pain therapy and finally to surgical procedures. Over the past decades, the number of spine surgeries has increased enormously [7]. This is partly because the number of otherwise healthy patients in our ageing society is increasing and better medical care is available, even in rural areas. Moreover, improvements in surgical, perioperative, and anaesthesia techniques have allowed a shift in the threshold of indications for surgery. On the other hand, some authors claim that over the past years, therapeutic strategies have overly focused on surgical treatment, an opinion that seems to be supported by an absolute increase in failure rates after lumbar fusions, followed by adjacent lumbar segment degeneration [8]. Lumbar spine surgery, with its complications, can be contrasted with conservative strategies that also achieve good results [9]. It is therefore of paramount importance to identify patients who will benefit from spine surgery. This is especially so in many cases in which patients’ complaints and medical history, together with clinical observations, do not correspond to structural pathological changes found on X-rays or magnetic resonance imaging (MRI).

For this reason, the concept of inpatient gradual diagnostics (IGD) has found widespread use in Germany over the past two decades [10]. Through local administration of analgesic and anti-inflammatory agents to a special area of interest, e.g. facet/sacroiliac joints or the epidural space, the effect of surgery can be temporarily simulated. From the patient’s statement about the alleviation of pain, the surgeon can draw conclusions concerning its cause.

The aim of this study was to evaluate the extent to which IGD influences the decision about the nature and scope of surgical treatment strategies, and the way in which it does so, in patients with chronic lumbar back pain.

## Methods

### Procedure

During our spinal consultation, we compare a patient’s clinical findings with radiographs (antero-posterior and lateral) and an MRI of the lumbar spine. In cases with a discrepancy between the clinical presentation and the radiological findings in particular, IGD is scheduled to evaluate the possible benefit to the patient from surgical intervention.

On the day that the patient is admitted for IGD, a laboratory blood analysis is performed to rule out possible infection. Over the course of the following days, the patient receives selective infiltrations at loci that, depending on the patient’s response to the anti-inflammatory and analgesic drugs, influence the decision of the ensuing treatment strategy. Target sites are mainly the facet joints, epidural space, sacroiliac joints, deep back muscles or the spinal nerve at its exit through the intervertebral foramen. In the case of an epidural injection, for safety reasons, patients are asked to stay in bed for 2 h with their trunk elevated 30°. Injections are performed daily and their effect is registered by repeatedly using the Numeric Pain Rating Scale before and after infiltration to assess pain and enquiring about pain alleviation as a percentage of pain reduction in the back or leg. The surgeon can thus draw conclusions as to the main causes of the patient’s pain. At the end of the week, from the synopsis of the responses to the injections, along with the clinical and radiological findings, a well-founded recommendation concerning future treatment is generated.

### Infiltration technique

Infiltrations are radiologically guided under a C-arm X-ray unit. The injection technique is based on the recommendations of Theodoridis and Krämer [11, 12]. The analgesic bupivacaine (1 %) and the corticosteroid triamcinolone (10 mg/ml) are injected at the site, the mixture and quantity of which varies, depending on the location being addressed.

### Evaluation of treatment strategy before and after IGD

All patients admitted to our department for IGD from January 2011 to December 2012 (*n* = 116) were selected for retrospective analysis and evaluated by two experienced spine surgeons. The medical letters generated during consultation before admittance to hospital describing the symptoms and the clinical findings were analysed, together with the radiologic images of each patient (X-rays and MRI; if MRI was contraindicated, then myelo-computed tomography was used). The surgeons were asked to determine the orthopaedic course of action they would recommend without the possibility of further evaluation by IGD. Eight weeks later, the same data were presented to the spine surgeons. This time, in addition, the medical reports created at discharge from hospital after IGD were made available. They contained the patients’ statements regarding the achieved effect by each of the infiltrations. The spine surgeons were then asked to determine the treatment strategy from the synopsis of all available data; however, they were asked to do this without knowing what their first assessment was for each patient.

### Statistical analysis

Distributions of variables within the study groups were assessed by histograms. Because age is non-normally distributed, it is reported as a median (range). Categorical variables are reported as absolute and relative frequencies. The graphic presentation is in the form of histograms and bar diagrams. Statistical analysis was conducted by using IBM SPSS version 22.

## Results

**Fig. 1:**
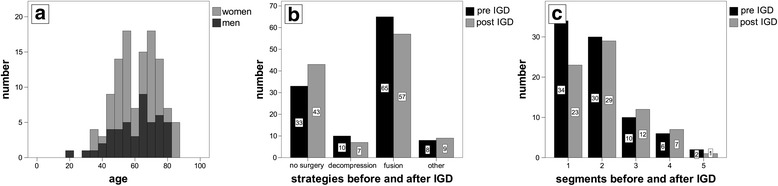
Analysed sample and treatment strategies. **a** Biometric data of the analysed sample. Two thirds of the patients undergoing inpatient gradual diagnostics (IGD) were female. Two major age groups of patients presenting themselves for IGD were observed: a first peak age at about the age of 50, consisting predominantly of women. A second peak age follows at about 70 years with a relatively higher percentage of male patients than at the first peak age. **b** The chosen strategy before and after IGD. **c** Scope of recommended procedures in terms of included segments in cases of a surgical approach

A total of 116 patients were analysed in this study. The median age was 63 years (20–87) and the ratio of men to women was 40:60 (Table 1). Two peak ages could be observed, with a rise in cases at about 50 years, and a second peak at about 70 years (Fig. 1a).

**Table 1:** Characteristics of the analysed sample and principal strategies before and after inpatient gradual diagnostics (IGD)

Characteristics	Total	Women	Men
Study group	116	70 (60 %)	46 (40 %)
Age, years	63 (20–87)	61 (35–87)	64 (20–82)
No surgery (pre/post IGD)	33/43	21/25	12/18
Decompression (pre/post IGD)	10/7	4/4	6/3
Fusion (pre/post IGD)	65/57	40/35	25/22
Other (pre/post IGD)	8/9	5/6	3/3

Without the information obtained during IGD, a surgical approach had been recommended for 83 of them. For the other 33 patients, conservative strategies had been favoured. A comparison of the recommendations given before IGD with those generated at the end of the inpatient diagnostics shows that the suggested treatment strategy had changed in 39 % of cases. In total, indications for surgery decreased from 83 to 73 cases (12 %), with more conservative approaches recommended instead (Table 2, Fig. 1b). This decrease in surgical procedures can be mostly attributed to a drop in monosegmental procedures, including both fusion and decompression surgery (Fig. 1b, c). The number of multisegmental indications changed only slightly. Of the initial 83 indicated operations, 18 (22 %) were rejected after IGD.

**Table 2:** Changes of treatment strategy after inpatient gradual diagnostics (IGD)

Treatment strategy	Number of cases/ Total cases	Percentage
Strategy changed through IGD	45/116	39
Strategy unaltered after IGD	71/116	61
No surgery indicated before IGD	33/116	28
Surgery indicated before IGD	83/116	72
Indication for conservative approach confirmed by IGD	25/33	76
Surgery indicated by IGD^a^	8/33	24
Indication for surgery confirmed by IGD	65/83	78
Indication for surgery refused by IGD^b^	18/83	22
Scope of initially suggested surgical procedure extended by IGD (without cases with no indication for surgery before IGD)^c^	17/65	26
Scope of indicated surgical procedure reduced by IGD (without cases with no indication for surgery after IGD)^d^	2/65	3

^a^Three two-segment fusions, three one-segment fusions, two one-segment decompressions

^b^In two cases, indication for total hip arthroplasty

^c^Extension of surgical approach: one segment cranially (*n* = 6), one segment caudally (*n* = 4), one segment cranially and one caudally (*n* = 1), change of decompression to fusion surgery (*n* = 5), monosegmental lumbar fusion in addition to removal of sacroiliac joint screws (*n* = 1)

^d^Change of fusion surgery to decompression (*n* = 1), reduction of fusion scope by two segments (*n* = 1)

**Fig. 2:**
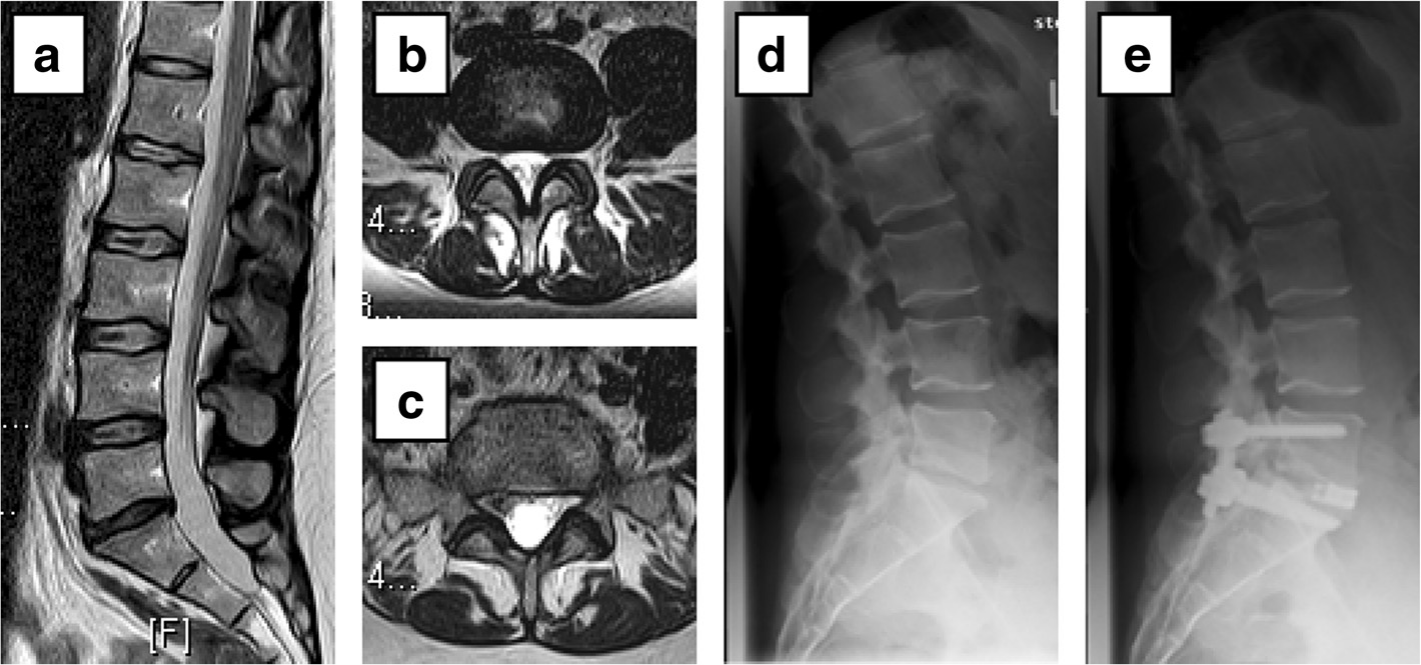
Confirmation of an uncertain indication for surgery. **a**-**c** T2-weighted magnetic resonance images preoperatively: **a** lumbar sagittal image and **b** axial lumbar image of L4/5 and **c** L5/S1. **d** Lateral radiographic image preoperatively and **e** postoperatively. The patient presented with chronic lumbar back and sciatic pain on the right side. Moderate intervertebral disc degeneration was observed between L5 and S1 (Pfirrmann grade 3-4 [13]) and a secondary neuroforaminal stenosis on the right side. Moderate facet joint degeneration was seen at L4/5 (Weishaupt [14, 15] grade 1 right and grade 2 left facet) and L5/S1 (Weishaupt grade 1 right, grade 2 left facet). Recommendation before inpatient gradual diagnostics (IGD): lumbar fusion of L5/S1 with transforaminal lumbar interbody fusion (TLIF). Results obtained during IGD: • epidural injection of L5/S1: 20 % sciatic pain relief. • bilateral facet joint infiltration of L5/S1: 80 % back pain relief. • bilateral sacroiliac joint infiltration: 10 % back pain relief. • iliac crest infiltration at muscular insertion of erector spinae: 80 % pain relief. Recommendation after IGD: lumbar fusion of L5/S1 with TLIF. At 1 year follow-up, the patient reported no pain

**Fig. 3:**
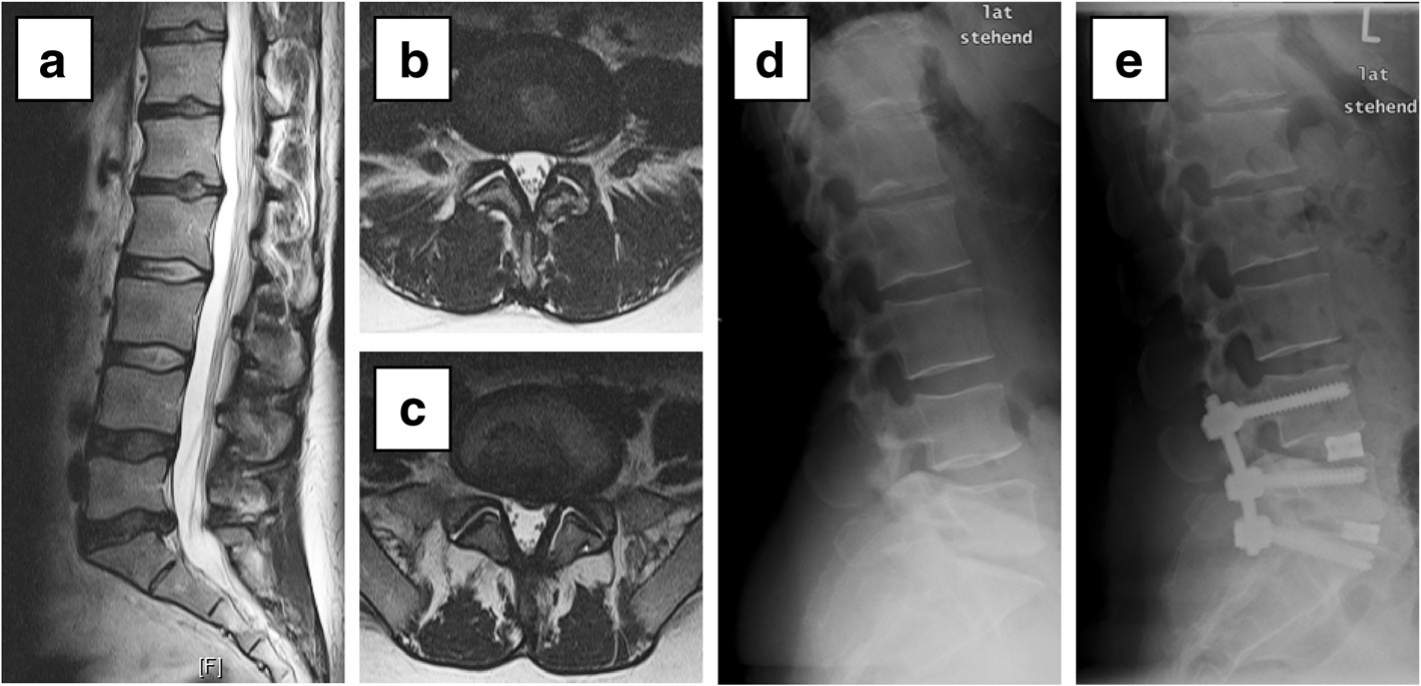
Extension of surgical scope by one motion segment cranially. **a**-**c** T2-weighted magnetic resonance images preoperatively: **a** lumbar sagittal image and **b** axial lumbar image of L4/5 and **c** L5/S1. **d** Lateral radiographic image preoperatively and **e** postoperatively. The patient presented with chronic lumbar back pain and sciatic pain on the left side and a sensorimotor deficit of 4/5 on the Medical Research Council scale for muscle power for ankle dorsiflexion, plantarflexion, and toe extension. Advanced intervertebral disc degeneration was observed between L5 and S1 (Pfirrmann grade 4) and a median intervertebral disc protrusion at L5/S1. Mild facet joint degeneration was seen at L4/5 (Weishaupt grade 0 right and grade 1 left facet) and almost no facet joint degeneration at L5/S1 (Weishaupt grade 0 bilaterally). Recommendation before inpatient gradual diagnostics (IGD): Lumbar fusion at L5/S1 with transforaminal lumbar interbody fusion (TLIF). Results obtained during IGD: • bilateral facet joint infiltration of L5/S1: 70 % reduction of back pain. • epidural injection of L5/S1: 40 % improvement of sciatic pain. • bilateral facet joint infiltration of L4/5: 60 % reduction of back pain. Recommendation after IGD: lumbar fusion of L4-S1 with TLIF. At 1 year follow-up, the patient reported no pain

**Fig. 4:**
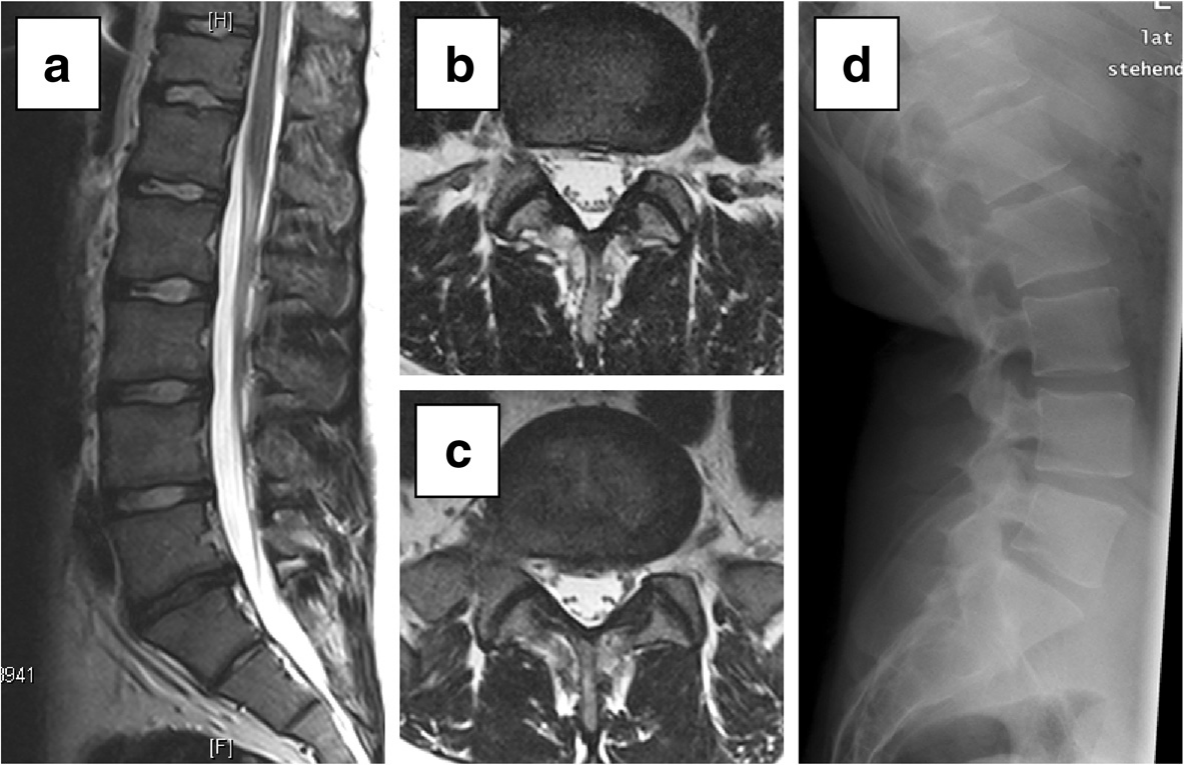
Recommendation for conservative treatment. **a**-**c** T2-weighted magnetic resonance images preoperatively: **a** lumbar sagittal image and **b** axial lumbar image of L4/5 and **c** L5/S1. **d** Lateral radiographic image. The patient presented with predominantly chronic lumbar back pain since childhood and intermittent bilateral sciatic pain. The patient works as a storeman. Advanced intervertebral disc degeneration was observed between L5 and S1 (Pfirrmann grade 4) and mild facet joint degeneration of L4/5 and L5/S1 (Weishaupt grade 1). Before inpatient gradual diagnostics (IGD), a lumbar fusion of L5/S1 with transforaminal lumbar interbody fusion had been discussed. Results obtained during IGD: • epidural injection of L5/S1: 0 % improvement of sciatic pain, 30 % back pain relief. • bilateral facet joint infiltration of L5/S1: 0 % improvement of back and sciatic pain. In view of the lack of response to the infiltrations, further conservative strategy was recommended. Half a year later, the patient had a discectomy of L5/S1 performed in another hospital, with initial good results for a few weeks, followed by a complete clinical relapse. When the patient thereupon presented himself in the department of neurosurgery of our hospital, our colleagues advised against further surgery, independent of our own recommendation

**Fig. 5:**
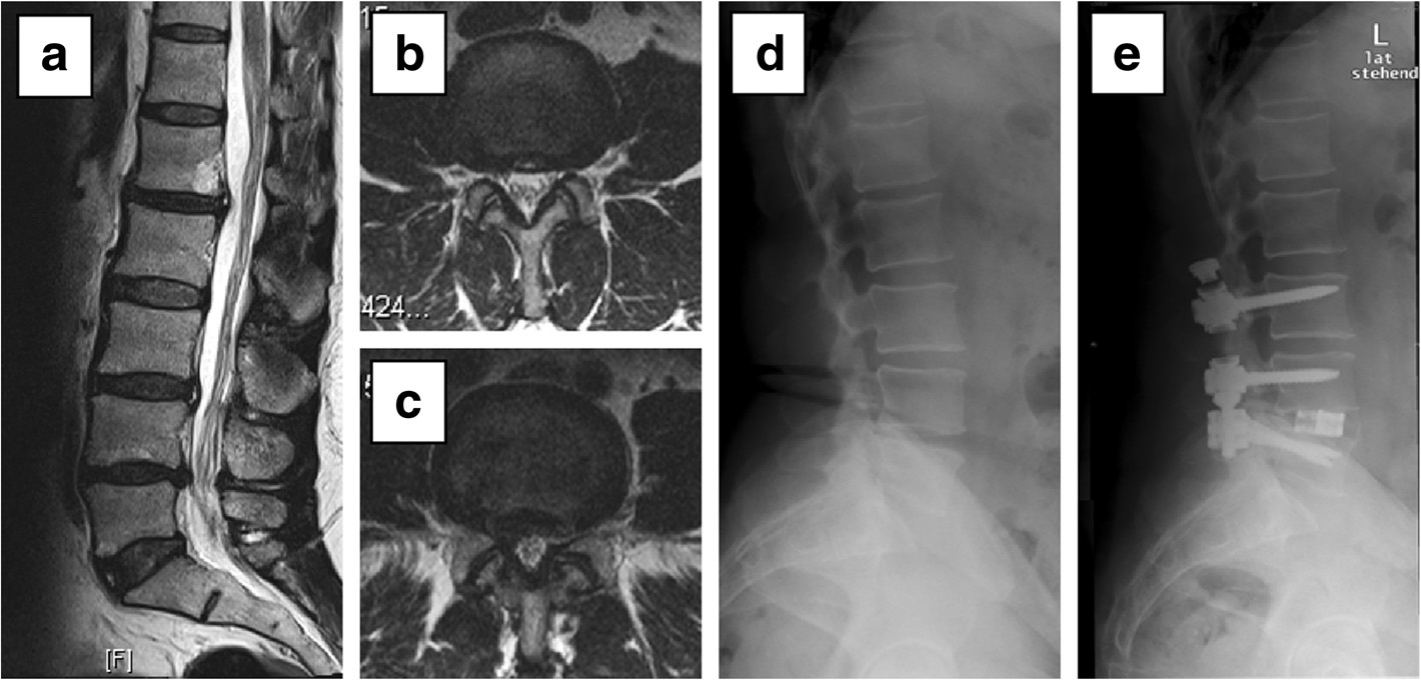
Semirigid inclusion of the cranial motion segment after IGD. **a**-**c** T2-weighted magnetic resonance images preoperatively: **a** lumbar sagittal image and **b** axial lumbar image of L3/4 and **c** L4/5. **d** Lateral radiographic image preoperatively and **e** postoperatively. The patient presented with chronic lumbar back, strong sciatic pain bilaterally, and a hypoesthesia at the right lateral lower leg. Moderate intervertebral disc degeneration was observed at L3/4 and L5/S1 (Pfirrmann grade 3), advanced degeneration at L4/5 (Pfirrmann grade 4-5), and a neuroforaminal stenosis on the right side due to facet joint hypertrophy and intervertebral disc protrusion (grade 2 according to Lee [16, 17]). Moderate facet joint degeneration was seen at L3/4 and L4/5 (Weishaupt grade 1). Recommendation before inpatient gradual diagnostics (IGD): lumbar fusion of L4/5 with transforaminal lumbar interbody fusion (TLIF). Results obtained during IGD: • bilateral facet joint infiltration of L3/4: 30 % pain relief back pain. • bilateral facet joint infiltration of L4/5: 30 % pain relief back pain. • epidural injection of L4/5: 30 % sciatic pain relief. • bilateral sacroiliac joint infiltration: 0 % pain relief. Although the absolute values for pain relief were only at moderate levels, the patient’s walking distance increased dramatically after the infiltrations. Recommendation after IGD: lumbar fusion of L3-5 with TLIF L4/5 and a semirigid instrumentation of L3/4. At the 3-month follow-up, the patient reported considerable pain relief and an increase in walking distance in comparison to that achieved preoperatively

At the same time, in about one quarter of these 33 patients in which a conservative approach had been initially suggested, after IGD, a surgical recommendation could be given. It is noteworthy that for the remaining 76 % of these patients (in which a possible surgical intervention had been taken into consideration although not recommended before IGD), a clear statement for conservative treatment and advice against surgery could be formulated after IGD. Changes is treatment strategy were comparable for both peak ages. Individual examples tare given in Figs. 2, 3, 4 and 5. Description of cases with a recommended procedure other than simple decompression or primary fusion surgery are in Additional file 1: Online resource 1.

## Discussion

IGD is an established concept for determining therapeutic strategies in patients with chronic lumbar back pain, especially in those cases in which symptoms do not correspond to radiomorphological findings. In addition, in multisegment degeneration, IGD is used to assess the necessary surgical scope. IGD leads to significant changes in recommendations for further surgical treatment. In summary, the total number of patients in the current study who were recommended to have surgery was reduced. The chosen operative scope, however, showed a tendency to be slightly more extensive than what would have been the case without IGD. This is, however, probably strongly dependent on the population of patients and the diagnoses that lead to indications for IGD. IGD does not have a therapeutic purpose in itself, but is designed to test different surgical target sites for their aptness for surgery. Pain alleviation is, therefore, only of short duration.

Regarding IGD as a concept, the extent to which local infiltrations can temporarily simulate the effect and success of spine surgery needs to be addressed. In our local situation, from our experience, success can be well predicted. For example, a patient with back pain caused by facet joint degeneration of L4/5 who has 100 % relief after facet joint infiltration has a high probability of benefiting from lumbar fusion of L4/5. On the other hand, we often do not know whether the pain is caused only by for example facet joint degeneration seen on MRI and X-rays. It is important to take into consideration that comorbidities such as mental instabilities or other diseases could strongly influence pain before and after surgery. This consideration is, however, much facilitated by IGD because of the intensive doctor-patient relationship and the observations drawn over the course of several days.

## Study limitations

The major limitation of this study is the fact that treatment strategies were determined retrospectively. It is possible that interaction with the patient personally might lead to a different recommendation, depending on the physician’s personal attitude towards spine surgery. Moreover, even though impressions of the patient’s mental and emotional condition are mentioned in our medical reports, a medical report can never replace direct doctor-patient contact. Finally, no intra- or interobserver correlation was performed but recommendations given as a consensus of two experienced spine surgeons. Future studies will have to address this aspect.

## Conclusions

IGD is an established concept used to determine therapeutic strategies in patients with chronic lumbar back pain. In our analysed collective, IGD led to highly relevant changes in recommendations for further surgical treatment.

## Abbreviations

ALIF, anterior lumbar interbody fusion; IGD, inpatient gradual diagnostics; L, lumbar vertebrae; MRI, magnetic resonance imaging; st. p., status post; Th, thoracic vertebrae; TLIF, transforaminal lumbar interbody fusion

## Additional files

Additional file 1: Online resource 1. Description of cases with a recommended procedure other than simple decompression or primary fusion surgery. (DOC 33 kb) Additional file 2: Online resource 2. Original data set describing evaluation results before and after IGD. (XLSX 36 kb)
