# Supplementary material for: Inpatient gradual diagnostics and its relevance for determining treatment strategies in lumbar back pain
Source: BMC Musculoskelet Disord. 2016 Jul 12;17:275. doi: 10.1186/s12891-016-1153-1 (PMC4941012; doi:10.1186/s12891-016-1153-1)
Supplement: Additional file 1: Online resource 1. — Description of cases with a recommended procedure other than simple decompression or primary fusion surgery. (DOC 33 kb) [file 12891_2016_1153_MOESM1_ESM.doc]

**Online resource 1. Description of cases with a recommended procedure other than simple decompression or primary fusion surgery**

| **Pre inpatient gradual diagnostics** | **Post inpatient gradual diagnostics** |
| --- | --- |
| Removal of metal implants from the sacroiliac joint, st. p. sacroiliac joint arthrodesis | Removal of metal implants from the sacroiliac joint and lumbar fusion of L5/S1 |
| No surgery, st. p. lumbar fusion of L4-S1 | Cranial adjacent segment fusion surgery of L2-4 |
| No surgery, st. p. lumbar fusion of L5/S1 | Dorsal reinstrumentation of L5/S1 and TLIF of L5/S1 |
| Dorsal reinstrumentation of L4/5 with cage revision of L4/5 (ALIF), st. p. lumbar fusion of L4/5 | Dorsal reinstrumentation of L4/5 with cage revision of L4/5 (ALIF) |
| Caudal adjacent segment fusion of L5/S1 and TLIF of L5/S1, st. p. lumbar fusion of L2-5 | Caudal adjacent segment fusion of L5/S1 and TLIF of L5/S1 |
| Cage revision of L5/S1, st. p. lumbar fusion of L5/S1 | Cage revision of L5/S1 |
| Cranial adjacent segment instrumentation of Th10-L1, st. p. lumbar fusion of L1-S1 | Cranial adjacent segment instrumentation of Th10-L1 |
| Caudal adjacent segment instrumentation of L3-S1 with TLIF of L4-S1, st. p. scoliosis fusion surgery in adolescence of Th4-L3 | Caudal adjacent segment instrumentation of L3-S1 with TLIF of L4-S1 |
| No surgery, st. p. lumbar fusion of L1-3 | Caudal adjacent segment instrumentation of L3-5, TLIF of L3-5 |
| Cranial adjacent segment instrumentation of L3/4, TLIF of L3/4, st. p. lumbar fusion of L4-S1 | No surgery |
| Craniocaudal adjacent segment instrumentation to L3 and S1 with TLIF of L3/4 and L5/S1, st. p. lumbar fusion of L4/5 | No surgery |

Abbreviations: st. p. - status post; ALIF - anterior lumbar interbody fusion, TLIF - transforaminal lumbar interbody fusion.
